# Characterization of Follicular Lymphoma in the Small Intestine Using Double-Balloon Endoscopy

**DOI:** 10.1155/2009/835258

**Published:** 2009-11-05

**Authors:** Manzurul Chowdhury, Masaki Endo, Toshimi Chiba, Norihiko Kudara, Shuhei Oana, Kunihiko Sato, Risaburo Akasaka, Kazumitsu Tomita, Saori Fujiwara, Tomomi Mizutani, Tamotsu Sugai, Yasuhiro Takikawa, Kazuyuki Suzuki

**Affiliations:** ^1^Department of Gastroenterology and Hepatology, Iwate Medical University, Morioka, Iwate 020-8505, Japan; ^2^Ministry of Health and Family Welfare, Government of Bangladesh, Polash, Narshingdi, Bangladesh; ^3^Division of Molecular Diagnostic Pathology, Department of Pathology, Iwate Medical University, Morioka, Iwate 020-8505, Japan

## Abstract

Follicular lymphomas occur rarely in the gastrointestinal tract, representing only 1–3% of all gastrointestinal tract B-cell non-Hodgkin lymphomas. We describe endoscopic analysis of 3 cases of follicular lymphoma in the small intestine using double-balloon endoscopy. Double-balloon endoscopy revealed multiple nodular lesions and elevated white patches, multiple polypoid lesions, and scattered white polypoid and nodular lesions in the duodenum and small intestine. Fuji Intelligent Chromo Endoscopy demonstrated small, whitish nodules, and narrow-band imaging showed a coiled, elongated vascular pattern within the elevated lesions. These cases are the first follicular lymphomas in the small intestine evaluated using narrow-band imaging or Fuji Intelligent Chromo Endoscopy to be reported.

## 1. Introduction

High-grade lymphomas are the most common histological subtype of lymphoma in the gastrointestinal tract, with the exception of the stomach, where low-grade lymphomas are more common [1–3]. Gastrointestinal lymphomas predominantly have a B-cell phenotype, whereas T-cell lymphomas are rare and usually arise in the small intestine [1]. Mucosa-associated lymphoid tissue (MALT) lymphomas are the most frequent type of low-grade non-Hodgkin lymphoma (NHL) encountered in the gastrointestinal tract. MALT lymphomas account for 40% of all primary gastric lymphomas, while multiple lymphomatous polyposis represents less than 10% of primary gastrointestinal lymphomas [4, 5].

Follicular lymphomas (FLs) represent a distinct type of low-grade NHLs characterized by neoplastic proliferation of germinal center B cells arranged in round aggregates that recapitulate the non-neoplastic germinal center. FL accounts for up to 22% of all NHLs, and up to 70% of all indolent NHLs [6–8]. Primary extranodal FL without peripheral lymphadenopathy is very uncommon. FL rarely occurs in the gastrointestinal tract, representing only 1–3% of all gastrointestinal tract B-cell NHLs [9–14]. Herein we report the endoscopic characterization of 3 cases of FL in the small intestine using double-balloon endoscopy.

## 2. Case Reports

**Figure 1:**
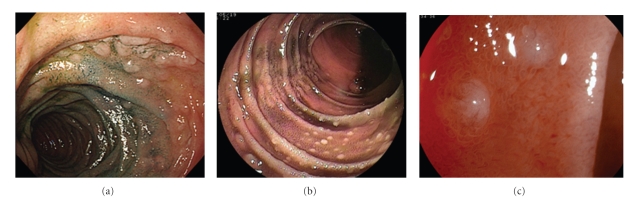
(a) Endoscopic view of the duodenum. Upper GI endoscopy revealed elevated white nodules in the second part of the duodenum. (b) Double-balloon endoscopic view of the jejunum. Double-balloon endoscopy showed multiple nodular lesions and elevated white patches in the jejunum. (c) FICE view of the jejunum. FICE showed whitish small nodules in the jejunum.

Case 1 was a 57-year-old male with a recent history of mild abdominal discomfort. Upper gastrointestinal endoscopy revealed white elevated nodules in the second part of the duodenum (Figure 1(a)). Biopsy specimens demonstrated FL that was positive for CD10 and BCL-2 and negative for CD5 and cyclin D_1_ on immunohistochemical staining. Double-balloon endoscopy showed multiple nodular lesions and elevated white patches from the jejunum up to the terminal ileum, with the majority of lesions concentrated around the proximal part of the ileum (Figure 1(b)). Fuji Intelligent Chromo Endoscopy (FICE) (EG590-ZW, Fujinon Toshiba ES Systems, Tokyo, Japan) revealed small, whitish nodules in the jejunum (Figure 1(c)). A computed tomography (CT) scan of the chest and abdomen showed involvement of para-aortic lymph nodes; thus, he was classified as having Stage II_2_ disease according to the criteria of the International Workshop in Lugano [15]. Although he was considered for chemotherapy, he died due to an unknown cause before treatment was initiated.

**Figure 2:**
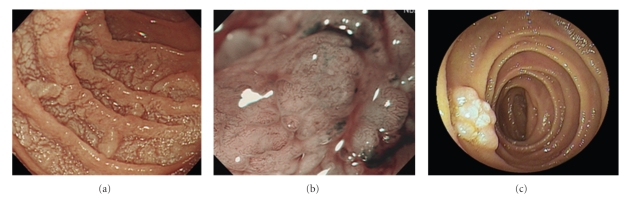
(a) Endoscopic view of the duodenum. Upper GI endoscopy showed multiple polypoid lesions in the duodenum. (b) NBI view of the duodenum. NBI demonstrated a coiled, elongated vascular pattern within elevated lesions of the duodenum. (c) Double-balloon endoscopic view of the jejunum. Double-balloon endoscopy showed multiple polypoid lesions in the jejunum.

Case 2 was a 66-year-old male who was found to have multiple polypoid lesions in the duodenum on routine surveillance endoscopy (Figure 2(a)). Narrow-band imaging (NBI) (H260Z, Olympus, Tokyo, Japan) demonstrated a coiled, elongated vascular pattern within elevated lesions of the duodenum (Figure 2(b)). Biopsy specimens revealed FL that was immunohistochemically positive for CD10 and BCL-2 and negative for CD5 and cyclin D_1_. Subsequent double-balloon endoscopy showed multiple polypoid lesions involving the second and third parts of the duodenum and the proximal part of the jejunum (Figure 2(c)). A CT scan of the chest and abdomen did not show an involvement of other structures or lymph nodes. Thus, the case was staged as Stage I according to the Lugano classification. As the patient was asymptomatic, he is currently undergoing regular observation.

**Figure 3:**
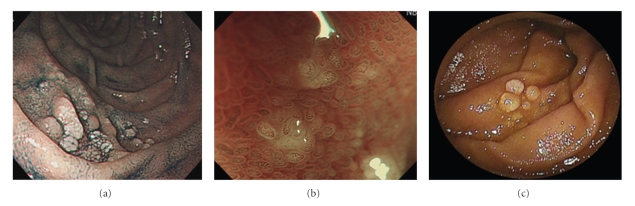
(a) Endoscopic view of the duodenum. Upper GI endoscopy showed a few scattered white polypoid lesions and a few isolated nodular lesions in the duodenum. (b) NBI view of the duodenum. NBI demonstrated a mildly elongated vascular pattern within elevated lesions of the duodenum. (c) Double-balloon endoscopic view of the jejunum. Double-balloon endoscopy showed scattered white polypoid and nodular lesion in the jejunum.

Case 3 is a 48-year-old asymptomatic male in whom a few scattered white polypoid lesions and a few isolated nodular lesions in the duodenum were identified during routine annual surveillance (Figure 3(a)). NBI demonstrated a mildly elongated vascular pattern within elevated lesions (Figure 3(b)). Biopsy specimens demonstrated the presence of FL, which immunohistochemical analysis revealed to be positive for CD10 and BCL-2 and negative for CD5 and cyclin D_1_. Subsequent double-balloon endoscopy showed scattered white polypoid and nodular lesions in the third part of the duodenum and the proximal part of the jejunum that were similar to the lesions observed in the second part of the duodenum (Figure 3(c)). A CT scan of the chest and abdomen showed a lack of other organ involvement. Thus, this case was also staged as Stage I according to the Lugano classification. He is currently undergoing regular observation.

## 3. Discussion

Double-balloon endoscopic analysis of early-stage disease has previously revealed multiple granules with a white color and rough surface in the duodenum and jejunum; multiple polypoid lesions resembling lymphomatous polyposis in the duodenum, jejunum, ileum, and rectum [16–18]; apparent lymphoid hyperplasia in the jejunum [19]. Capsule endoscopy of early-stage FL revealed patchy, whitish nodules with thickened mucosal folds in the proximal and middle parts of the small intestine [20], nodular lesions in the jejunum and ileum [21], and polypoid lesions in the jejunum [19]. The endoscopic appearance may vary from flat, elevated lesions to white, polypoid lesions, or a mixture of the two types. The polypoid lesions of FL may resemble lymphomatous polyposis or MALT lymphoma, both of which are associated with a poor prognosis [22]. Thus, there is a need to establish immunotyping and molecular biology assays to enable appropriate diagnosis, prognostic determination, evaluating prognosis, and treatment selection. Interestingly, an increased incidence of FL in the duodenum has been recently observed [10]. In the present analysis, 3 cases had lesions in the duodenum and jejunum, and 2 cases had lesions in the ileum.

Magnified endoscopy images of gastrointestinal FL have previously shown whitish granules corresponding to enlarged villi [16]. NBI is a novel endoscopic technique that can enhance the accuracy of diagnosis by using narrow-bandwidth filters in a red-green-blue sequential illumination system [23]. FICE simulates chromoendoscopy and can be used to evaluate microstructure and blood capillaries of the mucosal membrane [24]. The characterization of FL in the small intestine using the magnified endoscopy was clarified as a coiled or elongated vascular pattern within elevated lesions by NBI, and small or whitish nodules by FICE in our cases. Then, this is the first report to characterize FL in the small intestine by NBI or FICE.

Phenotypically, FL is characterized by expression of the pan B-cell antigens CD19, CD20, and CD22, while surface immunoglobulins, CD10, CD5, CD43, and nuclear cyclin D_1_ expression are generally not expressed. These features distinguish FL from MCL [12, 25–30]. In up to 85% of cases, the neoplastic B cells in FL differ from normal germinal center B cells in that they contain cytoplasmic BCL-2 protein [31–33]. B-cell BCL-2 protein expression is useful in discriminating FL from florid follicular hyperplasia, in which BCL-2 is not expressed.

Transformation of FL into diffuse, high-grade NHLs is relatively common and is associated with a poor prognosis [34], and the frequency of histological transformation is approximately 30% [35, 36]. The tendency of FL to transform into high-grade NHL highlights the importance of histological examination of even small lesions in the duodenum during diagnosis of primary FL of the gastrointestinal tract, as well as the importance of either double-balloon endoscopy or capsule endoscopy.

A randomized trial in FL that compared intensive therapy with a “watch and wait” strategy demonstrated no survival advantage for early intervention, thereby supporting the “Stanford philosophy” of expectant management unless treatment is perceived to be indicated [37]. Conventional treatment options for FL include surgery, chemotherapy, and radiotherapy. Recently, rituximab in combination with chemotherapy has been shown to result in high response rates in FL patients [38].

In conclusion, we have reported 3 cases of FL in the small intestine characterized by double-balloon endoscopy, which was also used to evaluate the accuracy of diagnosis of small intestine lesions. These cases are the first gastrointestinal FL cases evaluated by NBI or FICE to be reported.
